# The Welfare of Animals in Australian Filmed Media

**DOI:** 10.3390/ani11071986

**Published:** 2021-07-02

**Authors:** Peta L. Hitchens, Rachael H. Booth, Kirsten Stevens, Annabelle Murphy, Bidda Jones, Lauren M. Hemsworth

**Affiliations:** 1 Melbourne Veterinary School, Faculty of Veterinary and Agricultural Sciences, University of Melbourne, Werribee, VIC 3030, Australia; rbooth1@student.unimelb.edu.au; 2 School of Culture and Communication, Faculty of Arts, University of Melbourne, Parkville, VIC 3010, Australia; kirsten.stevens@unimelb.edu.au; 3 Victorian College of the Arts, Faculty of Fine Arts and Music, University of Melbourne, Southbank, VIC 3006, Australia; almurphy@unimelb.edu.au; 4 Sydney School of Veterinary Science, University of Sydney, Sydney, NSW 2006, Australia; bidda.jones@sydney.edu.au; 5 RSPCA Australia, P.O. Box 265, Deakin West, Canberra, ACT 2600, Australia; 6 Animal Welfare Science Centre, Faculty of Veterinary and Agricultural Sciences, University of Melbourne, Parkville, VIC 3010, Australia; lauren.hemsworth@unimelb.edu.au

**Keywords:** animal welfare, filmed media, Five Domains, animal actors, animal extras, Australian film, film industry, welfare assessment, animals in entertainment

## Abstract

**Simple Summary:**

Animals are frequently featured in film and television in Australia and globally. Monitoring and regulating animal welfare throughout production is therefore imperative for the film industry to maintain its social license to operate. In this commentary, we compare Australia’s state and territory-based legislation and regulation concerning the welfare of animals in filmed media to those in the United States and the United Kingdom and assess the regulations against the Five Domains Model of animal welfare. Historical examples of animal incidents in Australian film are used to illustrate deficiencies in regulation. We identify three themes of welfare concerns including incidents on-set, incidents off-set, and effects of portrayal on perception or ownership of specific species or breeds. A lack of uniform regulation across Australian states and territories is demonstrated, with regulations only partially addressing behavioural interactions or mental state of the animal. This highlights the need for standardised national legislation and improved monitoring and regulation of the welfare of animals in Australian filmed media.

**Abstract:**

Animals play a significant role in the production of film and television in Australia and globally. Given this, regulating and monitoring their welfare on- and off-set is imperative. We therefore aim to compare Australia’s state and territory-based legislation and regulation to those in the United States and the United Kingdom and assess regulations against the Five Domains Model of animal welfare. Historical examples of animal incidents in Australian film are used to illustrate potential deficiencies. We reviewed archived media for animal welfare incidents on and off production sets. We demonstrate a lack of uniformity, with 37.5% (3/8) of states and territories providing targeted Codes of Practice for animals in filmed media, and partially addressing behavioural interactions or mental state within the Five Domains Model. Three themes of welfare concerns were identified including incidents on-set, incidents off-set, and effects of portrayal on perception or ownership of specific species. This highlights the need for standardised national legislation and improved monitoring and regulation. Further research should quantify the number of animals used in productions, describe the type and duration of the work the animals undertake, investigate the frequency of animal welfare incidents, and explore alternative methods to the use of live animals in film and television.

## 1. Introduction

With the rise in animal activism and ethical consumerism, industries that use animals for entertainment are becoming increasingly scrutinised by the general public [1]. Animals play a significant role in the production of film and television [2,3], and thus these industries are at risk of losing their “social license to operate” (SLO) if they fail to demonstrate their commitment to safeguarding animal welfare [4,5]. Animals are used in different types of filmed media including movies, television series, theatre, commercials, promotional work, corporate training videos, photography, and music videos. They are also filmed for a variety of reasons including to add realism as background, as pets, companions, or sidekicks, or as a threat or foe [6].

Australia relies on state and territory-governed legislation and regulation to regulate the welfare of animals in filmed media including film, television, commercials and theatre. Codes of Practice (CoP) are implemented under the relevant Act and Regulations in each jurisdiction, and though they are not directly enforceable, they do provide a benchmark in which cases of alleged cruelty can be assessed under the relevant Act. Enforceable standards are in place for some animal use contexts through the regulation of Australian Animal Welfare Standards and Guidelines (e.g., [7]), but no specific Australian standard exists for the use of animals in filmed media. Other countries have different animal welfare regulation structures. 

Animal welfare regulation needs to be supported by standards that are assessable, enforceable, and based on the most recent scientific developments in animal welfare. The Five Domains Model of animal welfare assessment fits this brief [8,9] and has been applied to the assessment of animal welfare in broad settings including cases of animal cruelty [10], pest control [11], domestic horses [12,13], and in zoos [14]. The Five Domains Model, detailed in Section 2.2, is so named because it focuses on three internal survival-related factors (Nutrition, Physical Environment, Health), one external situation-related factor (Behavioural Interactions), and the overall affective experience factor that is combined in the fifth domain (Mental State), which also provides the opportunity for animals to have positive experiences, thereby supporting their optimal welfare [9]. This model was designed to facilitate standardised and comprehensive animal welfare assessments, with a focus on both welfare compromise and enhancement [8,15]. A limitation contributing to the difficulty and subjectivity in the interpretation of the Australian state and territory CoPs is that they are based on an older model. When these CoPs were created, the Five Freedoms Model, formulated in the early 1990s, was recognised as the animal welfare model of choice, incorporating freedom from hunger and thirst; discomfort; pain, injury, or disease; to express normal behaviour; and from fear and distress. Marked advancements in scientific understanding of animal welfare over the last few decades have highlighted the gaps in the Five Freedoms, particularly related to the elimination of negative experiences and the provision of more positive experiences [16]. The use of words such as ‘should’ and ‘recommended’ rather than ‘must’ and ‘mandatory’ also served to dilute the impact of such documents.

Most of the research conducted on the welfare of animals used for sports or entertainment relates to circuses [17], greyhound and horse racing [18], and other equine disciplines [19]; however, there remains a lack of research on the welfare of animals in filmed media. Currently, there is no published information pertaining to the species and number of each species used each year in Australian filmed media, the type and duration of the work the animals undertake on set, and the number of incidents such as illness, injury or death that occur. Understanding these factors is the first critical step in safeguarding the welfare of animals used in productions.

This commentary aims to highlight the need for animal welfare regulatory reform by contrasting Australia’s animal welfare regulations for animals in filmed media with regulatory bodies in the United States (US) and the United Kingdom (UK), and against the Five Domains Model for animal welfare assessment [8,9]. Issues identified are illustrated using historical examples of media-reported welfare incidents that have occurred on or off production sets in Australia. Although the ethical implications of using animals for entertainment is clear, this commentary is not intended to be a discussion about whether they should be used, rather, that adequate strategies are in place for their protection.

## 2. Regulation of Filmed Media and the Five Domains of Animal Welfare

Relevant animal welfare legislation and regulations for animals in filmed media in each Australian state and territory were sourced from the Australian Animal Welfare Standards and Guidelines website (www.animalwelfarestandards.net.au/; accessed on 1 March 2021). Additional information was gained by contacting each individual jurisdictional government department responsible for regulation of animal welfare. Regulations were then evaluated against the Five Domains Model for animal welfare assessment as a reference point (Table S1) [8,9]. Key aspects of regulations from Australia, the US, and the UK are discussed below.

### 2.1. Regulatory Structures of Filmed Media

#### 2.1.1. Australia

In Australia, there is no national legislation governing animal welfare. Each state and territory regulate animal welfare independently. Of the eight Australian states and territories, only three—Victoria, New South Wales (NSW), and the Australian Capital Territory (ACT)—have a CoP directly addressing the use of animals in filmed media [20,21,22]. Additionally, the Media Entertainment and Arts Alliance and Screen Producers Australia address some responsibilities when using animals in film under the Film and TV Safety Code 1983 [23].

Of the other Australian jurisdictions, Western Australia (WA) has no specific CoP for the use of animals in filmed media, but other CoPs may apply, for example, those addressing animals in entertainment including circuses, zoos and petting zoos, rodeos and racing, and exhibited animals [24]. In Queensland (QLD), a license or permit is required to exhibit most native wildlife or non-indigenous animals in film or television productions, but livestock and domestic animals do not require such licensing [25]. Jurisdictions also have CoPs that target the welfare of specific species, and these aspects would still apply to those being used in filmed media. This is the current situation for the Northern Territory (NT), Tasmania, and South Australia (SA). Further, the Australian Animal Welfare Standards and Guidelines for Exhibited Animals may be applicable, which set out enforceable standards and recommended guidelines, received national ministerial endorsement in 2019, and are in various stages of consideration across each jurisdiction [7].

#### 2.1.2. United States

In the US, there are no specific federal or state laws that govern the use of animals in film. Since 1940, the American Humane Association’s (AHA) Film and Television Unit, Humane Hollywood, has regulated the welfare of animals in filmed media. The AHA requires exhibitors and animals used in filming to be licensed by the United States Department of Agriculture (USDA) and reports the protection of ~100,000 animal actors by monitoring over 1000 productions per year. AHA Certified Animal Safety Representatives (CASR) monitor for breaches of the *Animal Welfare Act*, as well as compliance with the AHA guidelines [26]. 

The AHA ‘Guidelines for the Safe Use of Animals in Filmed Media’ are comprehensive, covering: veterinary care; productions, cast, and crew; reality programming, costumes, makeup, rigging, and props; location and set safety; special effects; stunts; and species-specific conditions [26,27]. However, for several reasons—namely industry sanctions, funding, awards, and certification—the regulation of animals in filmed media in America is arguably not independent. The AHA is authorised by the Screen Actors Guild–American Federation of Television and Radio Artists (SAG–AFTRA) and funded by the Producers Industry Advancement Cooperative Fund (IACF) to monitor and regulate animal welfare in filmed media [26]. However, AHA is not required to monitor non-SAG productions [27]. Further, abiding by the regulations ensures the film receives the “No Animals Were Harmed” certification [26]. This has been called into question following media reports of animal injuries or safety incidents in which films still received certification, for example, *War Horse* (2011) and *Life of Pi* (2012) [28]. Lack of independence in regulation can, and has, resulted in a lack of transparency, justification of policy decisions, and the perception of an impartial regulatory process that is skewed towards protecting the reputation of the productions rather than the welfare of the animals. Even just the perception of a conflict of interest can risk the industry’s SLO. 

#### 2.1.3. United Kingdom

In England, it is mandatory for companies that keep or train animals for exhibition to be licensed under *The Animal Welfare (Licensing of Activities Involving Animals) (England) Regulations 2018*. This Regulation was introduced as secondary legislation under the *Animal Welfare Act 2006*. Guidance for local authorities who are responsible for enforcing the licensing regime is available and modelled on the ‘RSPCA: Guidelines for the Welfare of Performing Animals’ [29]. However, these licensing conditions are less comprehensive and specific, focusing instead on how the animals are managed at their home facility, as well as not covering agents or productions that hire in animals where they do not directly own the animal, allowing ‘non-professional’ animals (i.e., pets) to often fall outside the system (pers. comms. Dr Ros Clubb, Senior Scientific Manager (Captive Wild Animals), RSPCA UK). Scotland and Wales continue to operate under the *Performing Animals (Regulation) Act 1925*. The RSPCA UK further acts as an independent advisory body, providing more comprehensive guidelines, albeit these guidelines are voluntary and do not have legal standing [29].

Within England and Wales, other legislation that may be relevant to the use of animals in filmed media includes, but is not limited to, the *Cinematograph Films (Animals) Act 1937*, *Dangerous Wild Animals Act 1976*; *The Welfare of Animals (Transport) (England) Order 2006*, *The Welfare of Animals (Transport) (Wales) Order 2007*, *The Welfare of Farmed Animals (England) Regulations 2007*, *The Welfare of Farmed Animals (Wales) Regulations 2007*, and the *Wildlife and Countryside Act 1981* (pers. comms. Dr Ros Clubb).

#### 2.1.4. Definition of Animals and Notification of Their Use

Table 1 presents the varying definition of animals under the relevant Animal Welfare Acts, Regulations, and CoPs. In Australia and the UK, animals are defined as any live member of a vertebrate species other than a human being [20,22,29]. In the US, under the US federal *Animal Welfare Act*, both live and dead ‘warm-blooded’ animal actors (e.g., mammals such as dogs, cats, primates) are protected, while cold-blooded animal actors (e.g., reptiles, amphibians) are not, though they are considered under the AHA guidelines, which although not legally protected, further encompasses any sentient creature including insects [27]. In England, regulations can be extended to include invertebrates under the Act if, on the basis of scientific evidence, such animals have the ability to experience pain or suffering. The RSPCA UK states in their voluntary guidelines that all animals, inclusive of invertebrates, for example, insects, crustaceans, and spiders, should be treated in the same respect though they are not legally protected [29]. This inclusivity in treatment should be considered under Australian regulations. 

In Australia, of the three jurisdictions with CoPs, only NSW requires the producer to notify the relevant agency that filming with animals will occur. In NSW, two agencies are named in the CoP, where either can be notified: RSPCA NSW and the NSW Animal Welfare League [20]. Where native animals are to be used, the producer may also be required to consult the NSW National Parks and Wildlife Service. In both the United States and the United Kingdom, monitoring of on-set action is voluntary and elected by the film producer. In the United States, the AHA requires registration of a production if it intends to use animals, but it is up to the production as to whether the animal action is monitored for compliance, and in order to earn the “No Animals Were Harmed” certification [8,9]. In England, it is mandatory for companies that keep or train animals for exhibition to be licensed [10], and thus if the company is licensed, notification of their use for individual productions is not required. Further, without the requirement to notify the regulating authority, productions using or abusing animals may not be identified until the production is released to the public or unless cast or crew come forward (i.e., whistleblowers).

### 2.2. The Five Domains Model for Animal Welfare Assessment

#### 2.2.1. Domains 1 to 3 (Nutrition, Physical Environment, Health)

The first three of the Five Domains involve survival-critical factors related to animal nutrition (availability of water and food), their physical environment (exposure to physical and atmospheric conditions), and health (injury, disease and physical fitness). As such, they are internal states brought about by external experiences; that is, modification of external factors, in which the animal has little to no agency, will affect the animal physiologically, pathologically, or clinically. These factors are measurable, which makes them relatively assessable in a welfare context compared to the latter domains [9]. Domain 1 (Nutrition) is largely covered by the regulations and CoPs (Table S1). However, inadequacies relating to Domains 2 (Physical Environment) and 3 (Health) are the most easily visible in our scenario, particularly if caused by exposing animal(s) to risks in their physical environment and/or inappropriate husbandry practices, training tools, or restrictive devices, any of which may be used unethically to elicit performance. Of the three Australian jurisdictions that directly address the use of animals in filmed media and in the US, all require the presence of a veterinarian for training, rehearsal, and filming or performances of scenes where there is a risk of distress or injury to the animals. Where there is minimal risk of injury or distress, veterinarians should be no more than 20 min from the site (or in the US, local enough to respond in case of emergency) [20,21,22,26]. Risky situations may include fast movement of any type by animals, large numbers of animals (e.g., racing, rodeo, battles, stampedes), very young, very old, or pregnant animals, obstacles required to be negotiated by animals, difficult terrain or ground surfaces, adverse weather, reduced visibility, or fire and/or smoke [20,26]. 

#### 2.2.2. Domains 4 and 5 (Behavioural Interactions, Mental State)

Whereas the first three domains primarily focus on internal survival-related factors due to external inputs to welfare, Domain 4 reflects the behavioural outputs caused by external situation-related factors and Domain 5 (Mental State) the emotional expression of these effects. These can be conveyed positively or negatively, or alternatively enhanced expression of agency vs. hindered expression of agency, that is brought about when animals interact with their environment, non-human animals, or humans [9]. This may be expressed by the animal through their behaviour (e.g., demeanour, level and type of activity, vocalisation). Animals used in productions may be subjected to a number of scenarios that result in sensory impositions, restricted choices, or constraints on activity or rest, and thus can be corrected if they are eliciting negative behavioural or mental states in the performing animal [8,16]. 

The Australian standards go some way to recognising these states as they also include provisions for species-appropriate enrichment with regard to cognitive, occupational, physical, feeding, sensory, and social enrichment [7]. The US AHA guidelines require observation for physical and behavioural changes that indicate discomfort [26], and the RSPCA UK guidelines state that animals should be allowed to express natural behaviours by providing, for example, environmental enrichment [29]. Negative effects on behaviour and mental state can last long after production is complete. For example, though the US AHA guidelines do recognise and raise issues surrounding the impact on primate socialisation and retirement plans (as apes for example can live up to 60 years yet typically retire from films at 8 years), adult non-human primates may still be worked on a closed set for up to 8 h per day, exclusive of transport time [26].

Training methods have also started to receive more attention, with the ACT CoP promoting positive reinforcement during training [21], the US AHA guidelines recommending that the animal trainer(s) sign an affidavit stating that they use only positive reinforcement techniques [26], and the RSPCA UK guidelines recommending the use of the most progressive and humane training methods (following the principles of positive reinforcement, rewarding desired behaviour) and providing advice on appropriate reinforcement and duration of training [29]. The Australian standards for exhibited animals (not specifically in relation to filming) provide a further precedent as they require that trained behaviours are reflective of those expressed by those animals in the wild [7]. This is in direct opposition to how animals have been used on set in the past, and thus films made that show animals in costume or conducting human-like behaviours, as primates are often required to perform, on-set may not comply. 

## 3. Historical Animal Welfare Incidents in Filmed Media

No published quantification of animal welfare incidents in Australian produced film and television product was identified. Due to the lack of other research, we sourced media articles citing historical animal welfare incidents occurring in Australian filmed media to demonstrate examples of deficiencies. We searched Google, Gale OneFile, and the Australian National Library of Australia’s Trove and Eresources portal for terms: (Animal* OR “Animal Actors”) AND (welfare OR “animal welfare” OR incidents) AND (film OR “filmed media” OR movie* OR entertainment OR video OR television OR theatre OR commercial* OR advertisement*) AND (Australia*). This was not intended to be a systematic review. Limitations of a media analysis in this context include that reporting of only severe incidents or those occurring on high-profile productions is likely. Categories were inductively derived from the incidents reported in the media articles to illustrate how regulation could be improved across these areas. These were (1) on-set incidents, (2) off-set incidents during breaks and transport, and (3) the influence of animals in filmed media. 

### 3.1. On-Set Incidents

On-set incidents that make it into the media are few and far between and predominately based on reports of injuries, illness, or death of animals falling under the third Domain (Health). In the case of the most recent incidents on *I’m a Celebrity…Get Me Out Of Here!* (UK program 2002 filmed in QLD, 2003–2019 in NSW; Australian program 2015–2020 in South Africa, 2021 in NSW; US program 2003 in NSW; German program 2004, 2008–2020 in NSW), there were reports of animals’ behaviour and mental state being negatively affected by their involvement in the show. On this reality television series, reptiles, rodents, and invertebrates are used in a series of trials. In Season 7 (2021) of the Australian version, two contestants were bitten: one on the face at least three times by a jungle carpet python snake (*Morelia spilota cheynei*), and another on the hand [31]. Over the years, the animals used have been “dropped, thrown, handled roughly, crushed, chased, overcrowded, scared by contestants and prevented from escaping from stressful experiences” [32]. In 2010, RSPCA NSW prosecuted the production company (ITV Studios) for committing an act of aggravated cruelty by the killing and eating of a rat on set.

Prosecutions for the two ‘celebrities’ involved were withdrawn following the company’s guilty plea [33]. The RSPCA has additionally expressed concern that there is potential for the general public to copy the ‘bushtucker trials’, whereby, for example, live invertebrates (e.g., cockroaches, crickets) are eaten by the contestants [32]. That the production company was prosecuted for harming a rat, but not for its treatment of reptiles and invertebrates, highlights the need for protection of the welfare of all animals, regardless of their species.

One of the most notorious animal welfare incidents in Australian film occurred on the *Man From Snowy River II* (1988) film set, where a catastrophically injured pregnant mare was killed via blows to the forehead using the blunt end of an axe [34]. The producer was later cleared of cruelty charges when it was determined that the mare was killed in the most humane way at hand, as no rifle or other method of humane euthanasia was readily available [35]. This particular situation would likely be avoided today, with veterinarians required to be on set when filming risky animal action. Horses, particularly, are subject to high risk when commonly used in fast action scenes.

Documentary or documentary-like footage used in narrative films has not always been under the same scrutiny. In the film *Wake in Fright* (1971), a scene showed genuine footage of a kangaroo hunt, which was edited to appear as a hunt within the film. Although professional licensed shooters were followed and filmed, the hunt went from 6 pm until past 2 am, at which point the kangaroos were wounded and killed inhumanely (allegedly due to the hunters being drunk). The director was open about the deaths, saying “some of the footage that I shot was so repulsive, heinous, and bloody that there was no way I could even use it” [36,37]. Similarly infamous, *Walkabout* (1971) featured several genuine hunting scenes that included the spearing and killing of a kangaroo and goanna, as well as the shooting of several buffalo. Due to these scenes, the film was reportedly blacklisted by the International Society for the Protection of Animals [38]. However, as the deaths were judged to be swift and as such did not “involve the cruel infliction of pain or terror on any animal”, they passed the standards set by the *Cinematograph Films (Animals) Act 1937* to allow for a UK release [39]. Only CoPs in NSW and AHA in the US explicitly include documentary under their definitions of filmed media, with AHA stating that scenes representing actual harm would be considered “exploitation of the animal’s suffering for the sake of entertainment”, and as such, depictions of harm must be simulated. Further, if wild animals in their natural habitat are to be filmed, they “should not be frightened, corralled, chased or otherwise manipulated” [26]. Despite these examples being from half a century ago, revelations about wildlife documentary footage being staged persist [40], and thus they should be subject to the same regulations as other filmed media. 

Likewise, *Bad Boy Bubby* (1993), a film shot in South Australia, came under fire from animal rights activists in Italy due to the film’s depictions of a distressed cat being dragged and tethered around the neck before being wrapped and strangled (implied) by clingwrap [41]. According to accounts from the film’s director and crewmembers, two live cats were used in the film with oversight from a veterinarian and representatives from the Animal Welfare League. One of the cats, a feral, was later euthanised in line with SA protocols for disposing of feral cats, with the dead cat also used for some shots. Despite the filmmaker’s assurances that no animals were harmed, some scenes were considered to depict genuine animal distress contravening the *Cinematograph Films (Animals) Act 1937* and were cut from the UK release [42]. Tightening of legislation and regulation in Australia to be in line with the UK is needed.

An American production being filmed in Australia calls into question who has jurisdiction. More recently, producers of *Pirates of the Caribbean: Dead Men Tell No Tales* (2017) applied to the federal Department of Environment for approval to import two White-throated Capuchin monkeys *(Cebus capucinus)* from California to QLD for filming. This was disputed by Humane Society International Australia, Wild Futures, Born Free Foundation and Captive Animals Protection Society with the argument that it was “cruel and unnatural”, that computer-generated images (CGI) could replace the primates in the film, and that their use may encourage the illegal wildlife trade [43]. RSPCA Australia also opposed the imports for additional reasons that (1) amending the list of species that may be imported under the *Environment Protection and Biodiversity Conservation Act 1999* sets a dangerous precedent for future applications, and (2) there are significant concerns for the welfare of the primates involving their use for entertainment purposes as well as the stress that confinement and transportation is likely to cause (pers. comms., Dr Bidda Jones, RSPCA Australia). Regardless, the application was approved, and the filming of the movie featuring the two capuchins went ahead. One of the capuchins reportedly experienced frequent vomiting throughout filming (the cause was not reported) and bit a make-up artist [44,45]. Despite this, the film was monitored by AHA CASRs and still received the “No Animals Were Harmed” full certification. 

The historical accounts of films that depict animal harm, death, and mistreatment on-screen and lack of research and objective data in this area makes it difficult to ascertain the prevalence of animal welfare incidents in Australian filmed media, or whether an enforcement gap, due to the lack of reporting, monitoring, and regulation has resulted in few documented incidents [46]. 

### 3.2. Off-Set Incidents

Animal welfare implications of the use of animals in filmed media are minimally addressed outside of film production (i.e., off-set). Animal welfare incidents occurring to animal actors and extras off-set may be even more difficult to monitor than those on-set. No reports of off-set incidents in Australia were identified. However, in New Zealand, in one of the most widely reported off-set incidents during production of *The Hobbit: An Unexpected Journey* (2012), twenty-seven animal actors including horses, chickens, sheep, and goats died in-between filming, largely due to inadequate housing and animal husbandry practices, including unsafe paddocks containing ‘bluffs, sinkholes and other “death traps”’. Though CGI accounted for more than half of all animals in this film, and the AHA monitored the animal actors on set, they were not monitored at other times [47,48]. This highlights the importance of looking after the welfare of the animal actors and extras at all times, not just during production. However, responsibility for the animals’ welfare needs to be clearly specified. In NSW, compliance with the CoP falls on the producer or their authorised agent, regardless of whether that person is on set [20]. In the ACT and Victoria, the responsibilities are with the producer(s) firstly, as well as the animal trainer(s) and veterinarian(s) [22], with the ACT additionally including director(s) and animal handler(s) [21]. 

### 3.3. Influence of Animals in Filmed Media

The media has reported increases in specific animal species or breeds being adopted, rescued, or acquired as a result of their appearance in popular film or television series. For example, following the release of *Finding Nemo* (2003), the media reported an increased popularity of clownfish (*Amphiprion ocellaris/percula*) as pets, coined ‘The Nemo Effect’ [49]. Subsequently, and because clownfish can be legally taken from parts of the Great Barrier Reef, Australian scientists developed a captive breeding program to keep up with clownfish demand in an attempt to quell the wildlife trade. Similar reports followed the release of *Finding Dory* (2016), though there was no evidence of an increase in imports of blue tang fish (*Paracanthurus hepatus*), at least in the US [50].

Several Australian films have anecdotally reported increases in specific dog breeds including Australian Kelpies following the release of *Red Dog* (2011), *Red Dog: True Blue* (2016), and *Koko: A Red Dog Story* (2019) [51], and one foster home reportedly rehomed more than 500 Maremma sheepdogs following *Oddball* (2015) [52]. There are no scientific studies demonstrating these increases in Australia; however, a US study observed new Dalmatian registrations increase sixfold from 6880 to 42816 over nine years following the re-release of *101 Dalmatians* (1985) [53]. Although Disney was warned of the potential for these dogs to become a ‘fad’ and implemented strategies to discourage irresponsible purchasing of Dalmatians, including the production of brochures on responsible pet ownership, this evidently had little effect [54]. Further, following the release of *Babe* (1995), the Rural Lands Protection Board reported a tenfold increase in the number of feral pigs in the bush surrounding Sydney, which was attributed to people buying piglets as pets following the film and then dumping the animals when they grew too large [55]. 

Media showing species in an entertaining or anthropomorphic light can change the public’s perception of that animal’s appropriateness as a pet. In Australia, exotic animals including primates and big cats can only be kept by licensed persons and cannot be sold for commercial purposes or kept as pets. In a US study, commercials featuring chimpanzees in human-like anthropomorphic situations resulted in viewers being less willing to donate to chimpanzee conservation and less aware of their inappropriateness as a pet when compared to those that viewed the control commercial of natural chimpanzee behaviour and a chimpanzee conservation commercial [56]. Similar findings were reported in other studies involving non-human primates [57] and big cats [58].

Conversely, some animals can be given worse reputations. In a secondary, but no less important issue raised on *I’m a Celebrity…Get Me Out Of Here!*, the program undertook a series of trials aimed at causing fear and discomfort to the human participants, with concern expressed by the RSPCA UK that the program deliberately portrayed certain species as nasty or frightening [32], for example, when participants were tasked with retrieving objects from a box full of overcrowded reptiles, rodents, insects, or arachnids such as spiders or scorpions. The different ways in which we anthropomorphise wild and domestic animals influences how humans interact with such animals, and in turn impacts those animals’ lived experiences [59]. Though ethical discourse is useful, animal welfare regulations should be based on evidence-based findings rather than popular media-constructed and often anthropomorphised narratives. Nevertheless, there is certainly a research bias towards domesticated mammalian vertebrates [60].

Reframing the way wild animals are depicted in filmed (and social) media, such as in educational documentaries, in films about their endangered status and their presence in the wildlife trade, may assist in changing the public perception of that species from appropriate as a pet to inappropriate [56,57,58]. Though the filmed media industries draw their discourse from various sources [54], they are responsible for its dissemination and therefore have a responsibility to ensure they depict animals in an appropriate manner.

## 4. Conclusions

This commentary highlights threats to the film and television industry’s social license to operate including exploitation of wild animals as actors, poor animal welfare outcomes, both on- and off-set, and inappropriate portrayal of species or breeds. We find Australia’s state and territory-based legislation, regulation, and the CoPs governing the welfare of animals in filmed media, lacking in uniformity and ineffective in monitoring and regulating animal welfare. 

A consistent nationwide approach to regulation, via a review and rewrite of national CoPs that are as comprehensive as those used in the US and UK, and that cover both on- and off-set conditions, is recommended. These CoPs should restrict the use of wild animals as actors and be monitored according to an objective framework such as the Five Domains Model. 

Furthermore, wherever possible, screen producers should consider production alternatives such as replacing the use of live animals with (for example) the use of CGI, the reduction of animal use in general, and/or the refinement of animal use, such as using positive reinforcement-based training methods or eliminating high-risk animal action shots. 

Reluctance to monitor and regulate animals in filmed media effectively may be due to lack of resources, but also because of the implications that increasing transparency might elicit, such as a push towards the full replacement of animals. This is not what we are suggesting here; rather, we are suggesting that transparency and a greater understanding of the issues will lead to better welfare outcomes for those animals involved. Both increased resources for the current animal welfare regulatory body (the RSPCA), and/or the establishment of clear national guidelines for the responsibility of monitoring and enforcement of the regulations, would further improve the existing inconsistent system. 

Further research is required to quantify the number of animals used in productions, to describe the type and duration of work the animals undertake on set, to investigate the frequency and type of animal welfare incidents in Australian film and television, and to explore alternative methods to the use of live animals. 

## Figures and Tables

**Table 1:** Definition of non-human ‘animals’ under the relevant Animal Welfare Acts, Regulations, Guidelines, and CoPs for filmed media across jurisdictions.

Animal Taxon	Jurisdiction						
	ACT ^a^	Victoria	NSW	US		UK	
				US *Animal Welfare Act* Code, 7 USC 2132(g)	AHA Guidelines	*Animal Welfare Act 2006*	*Performing Animals (Regulation) Act 1925*
**Vertebrates**							
Amphibian	[21]	[22]	[20]	nd	nd ^d^	[29]	[29]
Bird	[21]	[22]	[20]	[30] ^c^	[26]	[29]	[29]
Fish	[21]	nd	[20]	nd	[26]	[29]	[29]
Mammal	[21]	[22]	[20]	[30] ^c^	[26]	[29]	[29]
Reptile	[21]	[22]	[20]	nd	[26]	[29]	[29]
**Invertebrates**							
Cephalopod	[21]	nd	nd	nd	nd ^d^	nd ^d^	nd
Crustacean	[21] ^b^	nd	nd	nd	nd ^d^	nd ^d^	nd
Insects	nd	nd	nd	nd	[26]	nd ^d^	nd

nd = not defined; a—Animal Welfare (Animals Used on Film Sets) Code of Practice 2010 made under the Animal Welfare Act 1992; b—if intended for human consumption.; c—“…excludes (1) birds, rats of the genus *Rattus*, and mice of the genus *Mus*, bred for use in research, (2) horses not used for research purposes, and (3) other farm animals…”. d—not explicitly stated, but included if considered sentient or capable of experiencing pain or suffering. ACT: Australian Capital Territory; NSW: New South Wales.

## Data Availability

Not applicable.

## Supplementary Materials

The following are available online at https://www.mdpi.com/article/10.3390/ani11071986/s1, Table S1: A matrix of Australia’s states and territories’ regulations in comparison to the US and the UK regulations regarding the use of animals in filmed media and their incorporation of the Five Domains Model.
